# 

**Published:** 1889-06

**Authors:** 


THE ANNUAL MEETING OF THE AMERICAN MEDICAL EDITORS' ASSOCIATION will be held at the Casino, Newport, Monday evening, June 24, 1889. The programme includes the annual address by the President, Dr. W. C. Wile, editor *New England Medical Monthly*, subject: "Our Duty as Journalists, and the Reforms we should Persistently Advocate;" a paper by Dr. F. L. Sim, editor *Memphis Medical Monthly*, entitled "The Medical Editor's Relationship to the General Profession;" and one by Dr. T. D. Crothers, on "The Influence of Medical Journalism on the March of Science." The editor of the *New England Medical Monthly* and his associates, together with a few friends, will tender to the Association a steamboat excursion, and at the end of the sail an old-fashioned Rhode Island clam-bake will be indulged in. It is almost needless to add that the ladies are invited to this part of the entertainment.

The American Public Health Association will hold its Seventeenth Annual Meeting in Brooklyn, N. Y., Tuesday, Wednesday, Thursday and Friday, October, 22, 23, 24 and 25, 1889. The preliminary announcement gives a list of interesting topics for discussion, to which we shall refer again when we have more space to command.

### Literary and Other Notes.

THE ANNALS OF SURGERY, for May, 1889, has, as its leading article, a report by Dr. Geo. R. Fowler, of Brooklyn, of a unique case of an air tumor of the neck caused by a hernia of the pleura in a case of pneumothorax. It is well illustrated by a lithographic plate and by a photo-engraving. The editorial articles, which are always invaluable, take up the Topics of Injuries of the Heart, the Treatment of Cerebral Abscess, Cancer of the Larynx, and the Treatment of Enlarged Prostate by Electrolysis. The Department of Index of Surgical Progress contains an unusually copious and exhaustive series of classified abstracts of articles from foreign and domestic sources under about forty different titles. The usual number of book reviews conclude the number. The *Annals* continues to maintain its position as a publication of the first scientific rank, and is almost indispensable to every progressive practitioner.

WAMPOLE'S PREPARATIONS.—There are so many difficulties lying in the way of administering Cod Liver Oil, that it must frequently be put aside altogether in the treatment of wasting diseases, or malnutrition from any cause. Messrs. Henry K. Wampole & Co., of Philadelphia, have well-nigh solved this whole problem in furnishing to the profession a preparation in which the oil is so completely disguised as to taste, odor and appearance, that it can be administered to the most delicate person without producing nausea, or other unpleasant after-symptoms. The Extract of Wild Cherry, which this preparation contains, is of inherent value, besides its usefulness as a disguise. Wampole's Bromo-Pyrine is also taking a fast place in the armamentarium of the therapeutist. It should not be overlooked by any physician in casting about for a remedy to relieve brain-fag, headache, and nerve-tire in general.

THE COSMOPOLITAN is one of the best magazines that can be printed, and should find a place on every library table. Attention is called to the combination offer that is made to new subscribers on adv. page 16. The *Cosmopolitan* and the BUFFALO MEDICAL AND SURGICAL JOURNAL will be furnished to new subscribers for $3.00 a year.

### BOOKS AND PAMPHLETS RECEIVED.

An Elementary Treatise on Human Anatomy. By Joseph Leidy, M. D., LL. D., Professor of Human and Comparative Anatomy and Zoology in the University of Pennsylvania; President of the Academy of Natural Sciences, and of the Faculty of the Wagner Free Institute of Sciences, Philadelphia. Second edition, rewritten. With 495 illustrations, $6.00. Philadelphia: J. B. Lippincott Co. 1889. Buffalo: Peter Paul & Bro.

Cyclopedia of the Diseases of Children, Medical and Surgical. The articles written especially for the work by American, British and Canadian authors. Edited by John M. Keating, M. D. Vol. I., General Subjects, Fevers and Miasmatic Diseases. Illustrated; cloth, $5.00. Philadelphia: J. B. Lippincott Co. 1889.

Diseases and Injuries of the Ear—their Prevention and Cure. By Charles Henry Burnett, A. M., M. D., Aural Surgeon to the Presbyterian Hospital; one of the Consulting Aurists to the Pennsylvania Institution for the Deaf and Dumb, etc. Practical

Lessons in Nursing, Series. $1.00. Philadelphia: J. B. Lippincott Co. London: 10 Henrietta street, Covent Garden. 1889. Buffalo: Peter Paul & Bro.

Physiological Notes on Primary Education and the Study of Language. By Mary Putnam Jacobi, M. D. New York and London: G. P. Putnam's Sons. The Knickerbocker Press. 1889. Buffalo: Peter Paul & Bro.

The Ideals of the Republic; or, Great Words from Great Americans. Knickerbocker Nuggets. New York and London: G. P. Putnam's Sons. The Knickerbocker Press. Buffalo: Peter Paul & Bro.

The Story of Phœnicia. By George Rawlinson, M. A., Camden Professor of Ancient History in the University of Oxford, etc. New York: G. P. Putnam's Sons. London: T. Fisher Unwin. 1889. Buffalo: Peter Paul & Bro.

Diphtheria: Its Nature and Treatment. By C. E. Billington, M. D. Intubation in Croup and other Acute and Chronic Forms of Stenosis of the Larynx. By Joseph O'Dwyer, M. D. New York: William Wood & Co. 1889. Buffalo: J. H. Matteson.

A Manual of Diseases of the Ear, for the Use of Students and Practitioners of Medicine. By Albert H. Buck, M. D., Clinical Professor of Diseases of the Ear in the College of Physicians and Surgeons, etc. $2.50. New York: William Wood & Co. 1889. Buffalo: J. H. Matteson.

A Treatise on Hernia—the Radical Cure by the Use of the Buried Animal Suture. By Henry O. Marcy, A. M., M. D., LL.D., Boston, Mass. George S. Davis, Detroit, Mich. 1889.

Psychology as a Natural Science applied to the Solution of Occult Psychic Phenomena. By C. G. Raue, M. D. Philadelphia: Porter & Coates. 1889.

Transactions of the Dental Society of the State of New York. Twentieth Annual Meeting, Albany, N. Y., May, 1888.

The Relation of the Abdominal Surgeon to the Obstetrician and Gynecologist. By A. Vander Veer, M. D., Albany, N. Y. Reprint from *Gaillard's Medical Journal* for November, 1888. New York: The Judson Printing Co., 16 Beekman street.

Lactopeptine Medical Annual: The New York Pharmacal Association. 1889.

The Greater Half of the Continent. By Erastus Wiman, New York. Reprint from the *North American Review* for January, 1889.

The Resuscitation of Asphyxiated New-born Infants by the Suspension Method. By Llewellyn Eliot, M.D., Washington, D. C. Reprint from Transactions of American Association of Obstetricians and Gynecologists, September, 1888. Philadelphia: Wm. J. Dornan, printer. 1889.

Induction of Premature Labor. By Byron Stanton, M. D., Cincinnati, Ohio. Reprint from Transactions of American Association of Obstetricians and Gynecologists, September, 1888. Philadelphia: Wm. J. Dornan, printer. 1889.

The Question of Interfering with the Abscesses of Hip Disease. By A. B. Judson, M. D. Reprint from the *New York Medical Journal* for March 2, 1889.

Intubation of the Larynx in Diphtheritic Croup. Analysis of 200 cases operated upon. By Dillon Brown, M. D. Reprint from the *New York Medical Journal* for March 9, 1889.

Intubation in Chronic Stenosis of the Larynx, with a Report of Five Cases. By Joseph O'Dwyer, M. D. Reprint from the *New York Medical Journal* for March 10, 1889.

Views on the Prevention and Treatment of Typhoid Fever. Read before the Medical Society of the State of New York, February 5, 1889. By Stephen Smith Burt, M. D. From the *New York Medical Journal*, March 2, 1889. New York: Printed by Pusey & Co., 1398 Broadway.

Report of Sixty-three Cases of Alexander's Operation. By J. H. Kellogg, M. D., Battle Creek, Mich. Reprint from Transactions of American Association of Obstetricians and Gynecologists, September, 1888. Philadelphia: Wm. J. Dornan, printer. 1889.

Experimental Researches respecting the Relation of Dress to Pelvic Diseases of Women. By J. H. Kellogg, M. D. Reprint from the Transactions of the Michigan State Medical Society. 1888.

New Form of Posterior Colporrhaphy. By J. H. Kellogg, M. D., Battle Creek, Mich. Reprint from the *Boston Medical and Surgical Journal.*

The Question of Relationship between Lichen Planus (Wilson) and Lichen Ruber (Hebra). By A. R. Robinson, M. B., L. R. C. P. and S. (Edin.) Reprint from the *Journal of Cutaneous and Genito-Urinary Diseases* for January, February and March, 1889.

Pathology and Treatment of Alopecia Areata. By A. R. Robinson, M. B., L. R. C. P. and S. (Edin.) Reprint from the Transactions of the Ninth International Medical Congress. Vol. IV.

Announcement of the Thirty-first Annual Session of the Long Island College Hospital, Brooklyn, N. Y.

Merck's Bulletin: A Periodical Record of New Discoveries, Introductions, or Applications of Medicinal Chemicals. January, February, March and April, 1889. Published by E. Merck, Darmstadt, London and New York.

Address of Albert R. Baker, M. D., at the opening of the Medical Department of Wooster University, Cleveland, Ohio, Wednesday, February 17, 1889. Reprint from the *Cleveland Medical Gazette*, March, 1889.

Annual Address of the President of the Philadelphia Obstetrical Society. By Prof. Theophilus Pervin, M. D. Reprint from Annals of Gynecology. Boston, April, 1889.

Commercial Union with Canada. Speech of Hon. Robert R. Hitt, of Illinois, in the House of Representatives, Friday, March 1, 1889.

Cranial Measurements in Twenty Cases of Infantile Cerebral Hemiplegia. By E. D. Fisher, M. D., Adjunct Professor of Mental Diseases, University of New York; Professor of Nervous Diseases, Vermont Medical College; and Frederick Peterson, M. D., Chief of Clinic, Nervous Department, Vanderbilt Clinic College of Physicians and Surgeons; Attending Physician Hospital for Nervous Diseases, Blackwell's Island. Reprint from the *New York Medical Journal* for April 6, 1889.

A New Optometer. By Elmer Starr, M. D., Lecturer on Ophthalmology, Medical Department, University of Buffalo. Reprint from *American Journal Ophthalmology.*

The Extraction of Immature Cataract. Same.

Fees in Hospitals. By Henry J. Bigelow. From the *Boston Medical and Surgical Journal*, April 18, 1889.

The Galvanic Treatment of Fibro-Myomata. By A. H. Buckmaster, M. D., Assistant Surgeon to St. Peter's Hospital; Alumnus of the Woman's Hospital; Gynecologist to the Hospital for Mental and Nervous Diseases; Fellow of the New York Obstetrical Society. Prize Essay of the Alumni Association of the Long Island College Hospital for 1888. Published in the *Brooklyn Medical Journal*, November and December, 1888. New York: M. J. Rooney & Co., printers. 1888.

So-called "Varicocele" in the Female. By Henry C. Coe, M. D., M. R. C. S., Pathologist to the Woman's Hospital; Obstetrical Surgeon to Maternity Hospital; Assistant Surgeon to the New York Cancer Hospital. Reprint from the *American Journal of Obstetrics and Diseases of Women and Children*, Vol. XXII., No. 5, 1889. New York: William Wood & Co., publishers. 1889.

Bulletin of the Agricultural Experiment Station, Cornell University, College of Agriculture. V. April, 1889. I. On the Production of Lean Meat in Mature Animals. II. Does Heating Milk Affect the Quality or Quantity of Butter. Published by the University, Ithaca, N. Y. 1889.

New York Cancer Hospital, Eighth avenue and 106th street. Fourth Annual Report for the Year 1888.

The Printers and Mr. Childs. Celebration of the Birthday of George W. Childs. Banquet May 12, 1888. Philadelphia.

Second Biennial Report of the North Carolina Board of Health to the General Assembly of North Carolina. Session of 1889. Raleigh: Josephus Daniels, State printer and binder. 1889.

Fishing and Hunting Resorts of the Grand Trunk Railway. Compliments of Wm. Edgar, Esq., General Passenger Agent, Grand Trunk Railway.

Forty-third Annual Announcement of Starling Medical College, together with Catalogue and Order of College and Hospital Exercises for the Session of 1889-90.

Fourteenth Annual Announcement and Catalogue of Meharry Medical Department, Central Tennessee College, Nashville, Tenn., 1888-89.

HEALTH REPORTS.

Fourth Annual Report of the Board of Health of the City of Newport, R. I., for the Year 1888. Newport, R. I.: F. W. Marshall, printer. 1889.

Summary of Deaths in Newport, with Principal Causes, for the Year 1888, compared with those of 1887 and 1886.

Newport, R. I.: Report of Deaths and Contagious Diseases for the Months of March and April, 1889.

Monthly Bulletins of the New York State Board of Health, February and March, 1889.

Monthly Bulletin of the Tennessee State Board of Health, April and May, 1889.

Abstract of Proceedings of the Michigan State Board of Health, February 7, 1889.

Health in Michigan, March and April, 1889.

Weekly Abstract of Sanitary Reports U. S. Marine Hospital Service. Vol. IV., Abstracts Nos. 13, 14, 15, 16, 17, 18, 19, and 20.

### *DIGESTIVE FERMENTS IN THE INTESTINAL DISORDERS OF INFANTS.*

It seems somewhat strange, with our present knowledge of digestive ferments, that the application of pancreatin and pepsin in the diarrheas and intestinal disorders of children, especially those arising from inanition, is not more general.

We believe that an extension of the use of these products in such diseases would not only prove advantageous to the practitioner but save the lives of many little ones that otherwise would be doomed.

No practitioner, possessed of a modicum of therapeutical and physiological knowledge, will be found to admit that chalk mixtures, opiates, astringents, etc., meet fairly the indications in these cases. The anti-acids act merely mechanically, soothing the irritated mucous coat of stomach and intestines; the action of opiates which are especially dangerous to administer to nurslings, is uncertain, for it is impossible to gauge their use so as to attain the exact limit essential to intestinal anesthesia and arrest of peristaltic action, without narcosis; and astringents, while repressing secretion, at the same time retain and favor the absorption of ptomaines and other poisonous products which have provoked the flux—they limit the dejections at the expense of non-elimination of the toxic.

In such cases, and in all those of enfeebled digestion and in which the food remains undigested and fermenting in the stomach and intestines, pepsin and pancreatin and peptonized foods afford us pure and simple physiological remedies, whose administration is attended with no dangers; and their employment does not preclude the use of cathartics, or administration of antiseptics that are antitoxic to ptomaines.

Recently we have obtained the best results from such treatment, though it must be admitted in cases of unusual gravity, when collapse threatens, that cotto and wild yam are sometimes of value to check the flux, the digestive ferment following to secure proper digestion and nutrition. So long ago as 1856, Joulin and Corvisart (*Rev. Med. Chir. de Paris*) outlined this mode of treatment, and claimed the happiest results therefrom; and more recently it was advocated by Trousseauel, Pidoux, Barthez, and Rillet, of France, and Ellis and Davidson of the United Kingdom. Later still, Dr. J. Milner Fothergill (Handbook of Practice, p. 40,) remarks of pepsin: "Its utility in the treatment of imperfect digestion, and diarrhea in children, is certain." Prof. J. Lewis Smith (Prof. Dis. Children Bellevue Hospital Med. Coll.—Archiv. Pedriatic, 1886, p. 518,) expresses exactly the same opinion. Prof. Frederick John Farre (Parieras Mat. Med. and Therap., p. 943,) commends pepsin "*very highly in cholera infantum and summer complaints of children.*" And Bartholow declares (Mat. Med. and Therap., p. 68,): " Very great success has been attained in the treatment of the *diarrhea of infants* by pepsin. * * * The motions will be quickly changed in character, and the nutrition of the child improved, by giving it immediately after each supply of food." He further recommends (Naphey's Medical Therapeutics, p. 395,) the employment of peptonized milk or milk gruel for food in these cases, in which he is supported by Wilson Fox, (Diseases of Children, ii., p. 821,) who considers " pepsin invaluable in gastralgia and all irritative states of intestinal and stomach mucous membranes.

With such evidence, and with the physiological knowledge that at present obtains, it is evident that digestive ferments are too little studied or employed. Yet we must admit there have been good grounds for such neglect, in that the pepsins upon the market, for the most part, have been untrustworthy, and with no definite guide for testing, that of the U. S. P. being of a very low standard. These objections no longer obtain, however, for manufacturers have been led to provide accurate tests, and now disseminate the same in their literature. Thus we find Parke, Davis & Co. issue a work on Digestive Ferments, that is accurate in all details, and, further, they have placed upon the market a new pepsin of higher digestive power than any heretofore introduced, and possessing the exceptional advantages of being absolutely *free* from ptomaines, readily soluble, and of a digestive power hitherto unattained. Moreover, the standard of pepsin has been raised by the better manufacturers, and it is the practitioner's own fault if he is not able now to secure a preparation suited to his needs.

